# Biologic principles of minced cartilage implantation: a narrative review

**DOI:** 10.1007/s00402-022-04692-y

**Published:** 2022-11-16

**Authors:** Robert Ossendorff, Sebastian G. Walter, Frank A. Schildberg, Jeffrey Spang, Sarah Obudzinski, Stefan Preiss, Stefan Schneider, Gian M. Salzmann

**Affiliations:** 1 Department for Orthopaedics and Trauma Surgery, University Hospital Bonn, Venusberg Campus 1, 53127 Bonn, Germany; 2 Department for Orthopaedics and Trauma Surgery, University Hospital Cologne, Cologne, Germany; 3 Department of Orthopaedics, University of North Carolina School of Medicine, Chapel Hill, USA; 4 OrthoCentrum Hamburg, Hamburg, Germany; 5 Gelenkzentrum Rhein-Main, Hochheim, Germany; 6 Schulthess Clinic, Zurich, Switzerland

**Keywords:** Articular cartilage, Cartilage regeneration, Chondron, Minced cartilage implantation, Micronized cartilage, Cartilage fragmentation

## Abstract

Cartilage tissue has a very limited ability to regenerate. Symptomatic cartilage lesions are currently treated by various cartilage repair techniques. Multiple treatment techniques have been proposed in the last 30 years. Nevertheless, no single technique is accepted as a gold standard. Minced cartilage implantation is a newer technique that has garnered increasing attention. This procedure is attractive because it is autologous, can be performed in a single surgery, and is therefore given it is cost-effective. This narrative review provides an overview of the biological potential of current cartilage regenerative repair techniques with a focus on the translational evidence of minced cartilage implantation.

## Introduction

**Fig. 1:**
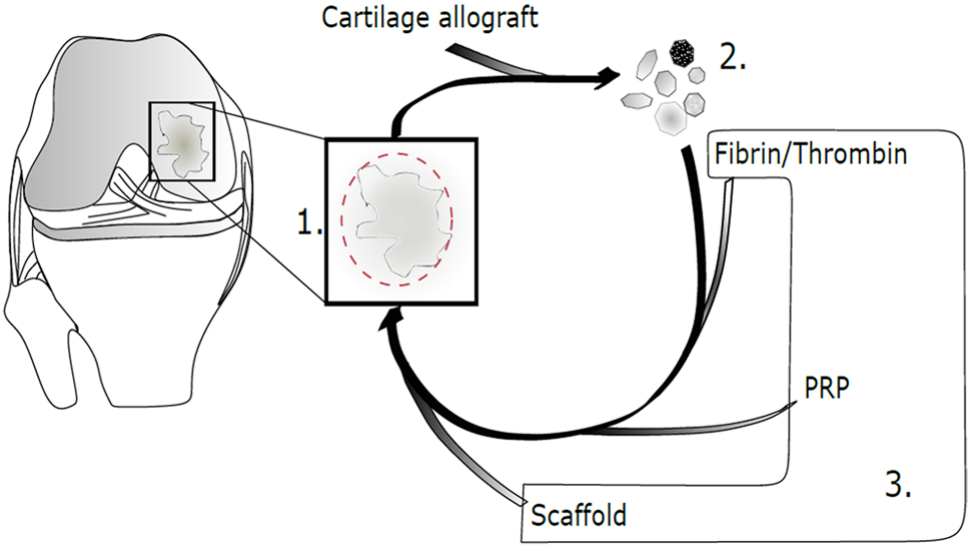
(1) Schematic of a medial condylar cartilage defect, which could be addressed by autologous cartilage implantation (ACI) or minced cartilage implantation (MCI). (2) For MCI, cartilage is harvested by hand with a scalpel or arthroscopically with a shaver from the edge of the defect area or from a non-weight-bearing region (e.g., intercondylar notch). In an arthroscopic setting, cartilaginous particles are collected through a drainage and a sequentially arranged sieve. Harvested cartilage is processed either manually by usage of a scalpel, a commonly used shaver or manufactured mincing devices. (3) MCI can be performed by different techniques such as addition of fibrin, a combination of fibrin and a scaffold (collagen, proteoglycans or synthetic), or addition of platelet rich plasma (PRP)—each yielding different biological effects. While fibrin supports tissue integration and cell migration, PRP inhibits catabolic effects of chondral degradation. Scaffolds are claimed to support tissue integration and could theoretically be loaded with growth factors

Over the years, various techniques have been described for the treatment of cartilage injuries [25]. No single cartilage repair technique is considered perfect for all applications. Concerning autologous biologic potency, autologous chondrocyte implantation (ACI) and osteochondral allografts are considered to be the gold standard, specifically ACI for large-diameter lesions [54]. However, ACI requires two surgical procedures, is expensive, and is highly regulated. In countries with routine access to allografts, fresh osteochondral allografts also have very good biologic healing potential. Alternatively, minced cartilage implantation is a single surgical procedure in which autologous cartilage is initially collected from the defect edge, minced into very small cartilage fragments, and then re-implanted for coverage of the defective area [59]. Fixation techniques of the cartilage fragments vary. Options include using autologous platelet-rich plasma, fibrin glue, membranes, or a combination of methods. Laboratory and animal studies have shown promising results using minced cartilage implantation for treatment of chondral and osteochondral lesions [18]. This is likely due in part to its biologic potential and the activity of the chondron, as well as enhancement via platelet rich plasma (PRP). The current clinical evidence supporting most minced cartilage implantation arthroscopically assisted techniques is not strong since it is a novel technique. This narrative review aims to provide an overview of the specific biologic mechanisms of the minced cartilage techniques and the potential of different co-treatments (Fig. 1).

## The chondron—functional characteristics

Articular chondrocytes are embedded within an abundant ECM that is composed of a crosslinked network of type 2 collagen, proteoglycans, and several important other collagens (e.g., 6, 9, 10, 11), and non-collagenous proteins. The ECM can be divided into pericellular matrix (PCM), territorial, and interterritorial regions. The PCM is a highly specialized, very thin layer of the ECM that immediately surrounds chondrocytes forming a functional unit together called the chondron [63]. This complex PCM is a host for a large variety of structures, including channels, receptors, and growth factors. The mechanical backbone is collagen 6. Though it has been shown that the PCM is mechanically much weaker when directly compared to the surrounding territorial/interterritorial matrix, [4] the PCM plays a major role in the metabolic activity as well as the mechanical signaling between the chondrocytes and the ECM. It also plays a critical role in cartilage homeostasis. For instance, PCM is very mechanosensitive and responsible for the transduction of a mechanical input into a biological signal [73]. Mechanical forces on the PCM activate specific pathways of gene expression (e.g., the Indian hedgehog gene pathway) which regulate chondrocyte differentiation and) [31]. Genetic upregulation provides a vital signal for chondrocyte production through determination and proliferation. Furthermore, the PCM acts in balancing anabolic and catabolic processes. It has been shown that chondrons in general express fewer catabolic enzymes (e.g., MMPs) under in vitro culture conditions than isolated chondrocytes [71]. The PCM also acts as a barrier. Chondrons react with significantly less volume change than individual chondrocytes when exposed to changes in osmolarity [37]. Additionally, chondrons have shown resilience to induced apoptosis in direct comparison to isolated chondrocytes. Zhang et al. investigated differences between chondrons and chondrocytes using a cDNA microarray of 200 different genes that are involved in chondrocyte proliferation, phenotype, and metabolism [79]. These authors found that chondrons upregulate the expression of type 2 collagen and aggrecan to a greater extent than chondrocytes when subjected to mechanical input [73]. Vonk et al. [71] suggested that preservation of the PCM results in positive effects on cell-induced cartilage production secondary to increased quantities of proteoglycans and decreased gene expression of MMP-13 in chondrons versus naked chondrocytes. Other growth factors have been identified as stored within the PCM which can be activated on-demand [47].

Changes to PCM properties in a pathologic state like OA may represent the disease state and also influence the regulatory function of the PCM. It has been shown that distorted cilia length and incidence are present with underlying OA [32]. This results in a disturbance in cilia-mediated signaling following alteration in mechanosensation and co-working ion channels. Such processes [28] activate serine proteases that again initiate PCM degradation, resulting in pathologic contact between robust collagen fibrils with the PCM and embedded chondrocytes [74]. Such contacted chondrocytes express catabolic enzymes (e.g., MMP-13) that in return degrade collagen 2 [76]. This feedback loop of serine proteases (HtrA1) to cell receptors (DDR2) and the MMP13 degradative pathway is one of the earliest events to occur in OA.

## Summary of functional characteristics of the chondron

- The PCM directly encapsulates the chondrocyte and forms a unit called the chondron.
- The chondron plays a major role in the metabolic activity as well as the mechanical signaling between the chondrocytes and the ECM
- PCM acts in balancing anabolic and catabolic processes
- Substantial structural changes in the PCM can be detected in OA

## The natural healing potential of articular cartilage and activated repair mechanisms

Articular cartilage is defined as a post-mitotic tissue; thus articular chondrocytes are not meant to divide post-puberty. Chondrocytes are responsible for extracellular matrix conversion in reaction to external stress [31]. This is a physiologic process capable of naturally repairing small lesions or allowing cartilage to withstand chronic overload with time. Yet, when an individual threshold (dependent on age, genetic disposition, musculoskeletal function, etc.) is breached, pathologic processes are initialized [35]. This is the origin of irreversible premature widespread osteoarthritis (OA) and surgical cartilage repair surgery may be indicated. Experimental evidence shows that loss of proteoglycans or alteration of their organization occurs before other signs of cartilage injury or effects on chondrocytes following impact loading [31]. Yet, with increasing trauma, the tissue damage also involves the chondrocyte on a cellular level. Intraarticular processes that lead to changes in the synovial fluid as well can negatively affect the articular cartilage and the chondrocytes that are embedded [46].

Mechanical causes of cartilage pathology fall into two general categories: (1) acute structural tissue damage induced by one intense load occurring at the instant of joint injury and (2) chronic overload related to malalignment or joint instability with gradual onset of structural damage and cartilage compositional degradation [26]. Chronic overuse or repetitive trauma results in continuous matrix breakdown. Presumably, the chondrocytes can restore the matrix if the proteoglycan loss does not exceed what the cells can rapidly produce, if the fibrillar collagen meshwork remains intact and if enough chondrocytes remain viable. When these conditions are not met, the cells cannot restore the matrix and the tissue will degenerate.

Chondrocytes respond to tissue injury by proliferating and increasing the synthesis of matrix macromolecules near the injury in an attempt at repair [11]. It is currently believed that mechanical load affects cartilage and joint breakdown progression through a wide range of molecular pathways. Overload activates its downstream molecular pathways of interleukin 1 beta (IL-1β), tumor necrosis factor alpha (TNF-α), nuclear factor kappa beta (NF-κB), Wnt, microRNA, and oxidative stress which induce apoptosis of chondrocytes and extracellular matrix (ECM) degradation [14, 26]. However, mechanical load does not always cause harmful effects on the articular surface since the physiological mechanical load can activate the TGF-β pathway to protect the cartilage. Many receptors detect mechanical signals in articular cartilage including integrins, ion channel receptors, Hic-5, Gremlin-1, and TRPV-4 [26]. These molecular mechanisms provide potential targets for clinical prevention and treatment of OA induced by mechanical load.

In direct surface impaction injuries, acute contusion of the cartilage may or may not be associated with clinically detectable articular surface fracture even though there may be significant cell death. Acute cell death can then spread throughout the tissue adjacent to the initial injury site, resulting in enlargement of the lesion. Simultaneously, matrix breakdown occurs, further limiting the potential for repair. This process has been demonstrated in several different studies. Work carried out by Tew and group [66], for instance, demonstrated that wounding cartilage with trephine results in necrotic cell death immediately adjacent to the lesion edge followed by a ‘wave’ of apoptosis extending into the nearby tissue from the lesion edge. Directly following, in 2004 Redman [57] comprehensively described the natural responses of articular cartilage to sharp and blunt trauma. By use of a translational in vitro experiment it was shown that in the case of blunt wounds, a band of cell death was observed adjacent to the lesion edge. Microautoradiography demonstrated little radiolabeled incorporation and, therefore, no new matrix synthesis or cell proliferation within this region. In contrast, wounds made with a sharp scalpel showed restricted cell death, with radiolabel incorporation adjacent to the lesion edge at all time points. This demonstrated not only chondrocyte proliferation and new matrix synthesis at the wound margin, but also an up-regulation of matrix synthesis adjacent to the lesion edge. In 2006, Lu et al. [45] reported the potential of minced cartilage to aid in cartilage healing. They found that cartilage particulation mobilizes matrix-embedded chondrocytes via increased tissue surface area. The in vitro outgrowth study indicated that fragmented cartilage tissue is a rich source for chondrocyte redistribution. The chondrocytes outgrown into 3-D scaffolds also formed cartilage-like tissue when implanted in SCID mice. In the same study, direct treatment of full-thickness chondral defects in goats using minced cartilage on a resorbable scaffold produced hyaline-like repair tissue at 6 months. Yet, Wang and co-workers [72] directly compared chondrogenic expression under the influence of mechanical stress between isolated chondrocytes, artificial matrix-encapsulated chondrocytes, and chondrons. While it was shown that encapsulation had beneficial effects the chondron outperformed the other groups concerning the production of hallmark articular cartilage genes Collagen 2 and Aggrecan [73]. It has also been shown that native articular cartilage pericellular matrix (PCM) and ECM components provide signals to drive undifferentiated cells toward chondrogenesis [16]. Other synergistic pathways between chondrocytes/chondrons and chondrogenic differentiation of ubiquitous mesenchymal stromal cells (MSC) may also play a role in the viability of minced cartilage procedures [7].

## Summary of the natural healing potential of articular cartilage and activated repair mechanisms

- Chondrocytes are responsible for extracellular matrix production in reaction to external stress
- A threshold specific to each individual determines whether osteoarthritis develops
- Acute cartilage trauma and chronic overload result in different molecular conditions
- Postmitotic human adult chondrocytes can be activated by particulation
- Activation results in proliferation and extracellular matrix production

## Biologic effect of the mincing process

The surgical technique of cutting cartilage into small pieces to induce a cartilage repair response was initially reported by Albrecht et al. 1983 in a rabbit model [3]. Cartilage defects of 23 knee joints were left untreated, whereas 52 joints were treated with small cartilage fragments and fibrin glue. After a follow up of 40 weeks, a histological assessment of the treated group showed hyaline-like cartilage. In the control group, no hyaline-like tissue was detected. Cartilage fragmentation induces reparative processes by activation of chondrocytes in migration, proliferation, and differentiation [43, 45]. The aim of the mincing procedure is to cut vital cartilage fragments with an optimized biologic potential to create neo-cartilage. Nevertheless, the cutting procedure can affect chondrocyte viability. Redman et al. [57] reported that blunt trauma significantly increased cell death compared to sharp trauma. A sharp instrument (scalpel, shaver, mincing device) is necessary to avoid the negative effects of cutting. Levinson et al. [43] investigated in an in-vitro study chondrocyte viability and outgrowth behavior of different mincing devices. The mincing device resulted in a higher outgrowth, but chondrocyte viability was equal to scalpel preparation. Moreover, the size of the fragments plays an important role. The smaller the fragments, the higher the potential for proliferation and differentiation of chondrocytes [8]. Bonasia et al. compared the biologic potential of four different sizes of human cartilage fragments in-vitro (8 mm × 0.3 mm, 2 × 2 mm, < 0.3 mm × 0.3 mm). They performed the mincing procedure with a scalpel. The group with the smallest fragment size and paste-like structure showed the highest ECM production after 6 weeks in culture. Lei et al. [42] reported in an in-vitro culture system of rabbit cartilage that small fragments (0.5 × 0.5 × 0.5 mm) demonstrated higher chondrocyte outgrowth compared to cartilage chunks (4 × 4 × 1 mm), which was furthermore associated with a significantly higher expression of membrane type 1-matrix metalloproteinase after 2, 4 and 6 weeks of culture. Marmotti et al. investigated that the outgrowth of rabbit cartilage fragments could be additionally enhanced by an injectable hyaluronic acid scaffold [49]. It remains a matter of debate which fragment size is the best for the biologic potential and the viability of cartilage fragments. To our knowledge, there is a lack of in-vivo studies comparing different fragment sizes. Furthermore, mincing is widely performed with a shaver. The reported advantage of a shaver compared to a scalpel is a faster mincing procedure and more homogenous fragmentation of the cartilage [61]. Nevertheless, there is currently no study available comparing between different shavers and the use of scalpels. Further studies need to address the questions: What are the consequences of shredding the cartilage with a shaver regarding chondrocyte viability and outgrowth? Are there differences compared to scalpel mincing? What is the best shaver type?

An important fact of consideration is the age-dependent biologic potential of cartilage fragments. Chondrocytes of minced cartilage from the inter-condylar notch of young patients undergoing anterior cruciate ligament reconstruction (ACLR) (mean age 24.3 years) demonstrated a higher migration potential compared to subjects undergoing prosthetic joint interventions (age 50–70 years) [51]. The response to growth factors was lower in cartilage fragments of older patients. On top, with increased age, a reduced proteoglycan synthesis and calcification of the tissue can lead to a lower chondrocyte migration and differentiation potential [9, 24]. Adkisson et al. [2] reported a 100-fold higher content of proteoglycans in juvenile neocartilage compared to adult neotissue. A significantly higher chondrogenic mRNA expression profile was detected in neo-cartilage of the younger group, which underlines the stronger potential of juvenile cartilage. Furthermore, the inflammatory surroundings with elevated cytokine levels can also affect chondrocyte viability and outgrowth potential [15, 56]. Hamasaki et al. [34] demonstrated in a murine co-culture model of macrophages and cartilage fragments a release of proinflammatory cytokines (TNFα, IL-6) resulting in catabolic effects with upregulation of matrix-metalloproteinases.

Allogeneic chondron transplantation is attractive due to the access to fresh juvenile allografts, which have strong biologic potential. There is evidence in-vitro and in- vivo that allogeneic transplants are safe and effective [18]. Bonasia et al. [10] reported a stronger biologic effect by combining autologous adult and juvenile allogeneic chondrons, compared to a single autologous adult chondron treatment. The commercial juvenile allograft product DeNovo NT of donors in a range from neonates to 13 years is commercially available in North America and recommended to be implanted prior to 40–45 days after harvest [77]. Longer storage was reported to negatively affect tissue quality.

Biologic potential is dependent on the harvested area. The highest regenerative capacity was reported from the healthy cartilage from the edge of the defect area, and non-weight bearing regions were inferior [6]. The biologic potential was higher in the edge of the defect compared to the central defect area of human cartilage fragments [1].

## Summary of the biologic effect of the mincing process

- Biologic potential is dependent on the fragment size (smaller fragments have a stronger potential)
- Cutting with sharp instruments is important for a high viability
- Chondrons of younger donors have a higher regenerative potential
- Allogeneic and autologous procedures are available

## Requirement for matrices in cartilage repair

Any cartilage repair procedure aims to generate the best possible hyaline cartilage-like tissue. Different strategies have been implemented in the field of tissue engineering and regenerative medicine (TERM) to provide a three-dimensional surrounding to optimize the conditions for chondrogenesis [40]. Nevertheless, the resulting tissue produced through these techniques is still not equivalent to the original cartilage. Scaffold structure determines the mechanical properties, cell attachment, proliferation, and differentiation of the seeded chondrocytes and the resulting neo-tissue [62].

Among the natural scaffolds, collagen scaffolds are attractive because of their high biocompatibility, biodegradability, and chondrocyte adherence [38]. Collagen scaffolds are typically used in third generation autologous chondrocyte implantation (MACI) and autologous matrix-induced chondrogenesis (AMIC) with excellent clinical results in short- and long-term follow-up [12, 70]. In clinical application of the second generation minced cartilage repair technique, a collagen membrane can be used in combination with fibrin glue to provide 3D surroundings for cartilage regeneration and a barrier to avoid cell diffusion [52]. Chaipinyo et al. [13] reported maintenance of chondrocyte differentiation and proliferation in collagen 1 gel scaffold constructs using an in vitro model of human fragmented cartilage. Levinson et al. [43] performed an in vitro comparative study of minced cartilage in fibrin versus collagen hydrogel. Both biomaterials resulted in equal histological outcomes regarding chondrocyte outgrowth and survival. Interestingly, matrix deposition was not enhanced by scaffold implementation. Lind et al. [44] investigated a goat model of minced cartilage implantation vs. autologous chondrocyte implantation with a collagen 1 scaffold (Chondrogide^®^, Geistlich). Minced cartilage was placed in the defect covered by the collagen membrane, while cultured chondrocytes were seeded onto the membrane. There was no histologic difference in O’Driscoll and Pinada score during 4 months follow-up. Massen et al. [52] evaluated clinical outcomes after minced cartilage implantation (MCI) with the combination of collagen scaffold and fibrin glue in a case series of 27 patients with a 2-years follow-up. They reported a significantly decreased pain and increased function score. Radiographic analysis six months postoperatively (MOCART score) did not show a full defect integration, but the outcome was in the range of other cartilage repair techniques. Matsushita et al. [53] investigated a comparison rabbit model of minced cartilage versus autologous chondrocyte implantation (ACI). The minced cartilage technique showed good regenerate quality using cells and fragments embedded in atelo-collagen. Tsuyuguchi reported similar results of this scaffold type in a preclinical model [67].

Hyaluronic Acid (HA) is an important component of cartilage ECM for lubrication and is frequently used as hydrogel to provide 3D conditions for cartilage regeneration. This provides a porous yet solid scaffold. A disadvantage of these natural polymers is their weak mechanical properties. Marmotti et al. [51] investigated a comparison study of hyaluronic acid (HA) derived scaffold vs. an HA-derivate membrane scaffold. Preloading with growth factors TGF-β1 and G-CSF increased the outgrowth and proliferation potential of human cartilage fragments of young and adult donors. Additionally, using rabbit models, they compared MCI in HA scaffold composite combined with fibrin glue versus PRP. They found the combination of HA with PRP resulted in the best repair tissue at 6 months. Fibrin glue decreased cartilage regeneration potential [49].

Synthetic scaffolds have also been implemented in MCI. Lu et al. [45] investigated the chondrogenic potential of minced cartilage in a synthetic biodegradable polyglycolide/polylactide (PGA/PLA) scaffold with a polydiaxone (PDS) mesh in a goat model. They demonstrated a good chondrocyte outgrowth of the implanted cartilage fragments. Histologic evaluation at 6 months showed hyaline-like tissue with a good integration compared to scaffold-only or empty control [45]. Frisibie et al. [29] investigated an equine model of a cartilage autograft implantation system (CAIS) versus autologous chondrocyte implantation (ACI) in a polydioxanone-reinforced foam scaffold. CAIS resulted in the highest histological and immunohistochemical scores. Cole et al. [20] performed a randomized controlled trial of a CAIS vs. microfracture minced cartilage combined with fibrin glue on a synthetic, absorbable scaffold and fixated with a mesh [20]. Clinical scores IKDC and KOOS were significantly increased up to 24 months following surgery in the synthetic scaffold group.

Nevertheless, while each scaffold has individual positive and negative characteristics all scaffolds share some drawbacks in scaffold handling. Ciglic et al. [19] investigated the impact of sutures on cartilage regeneration, which are often necessary to generate a safe graft fixation. Trans-articular sutures induced an acute cartilage injury with thickness about twice that of the thread. Less compressible monofilament PDS suture increased the tissue injury compared to softer braided Vicryl. Moreover, Hindle et al. investigated that cell viability decreased to 28.8% after crushing scaffold material with forceps in a viability study of membrane-induced autologous chondrocyte implantation (MACI) [36]. Currently, most surgical techniques of minced cartilage implantation are performed without the use of scaffolds.

## Summary requirement for matrices

- Scaffolds are implemented to support tissue growth and integration of chondrons in minced cartilage implantation
- Collagen 1 scaffolds are the most common type but do not represent the primary natural tissue component of hyaline cartilage
- In vitro and in vivo studies of MCI show good quality of the regenerate by scaffolding with different synthetic and natural scaffold types
- Scaffold free techniques are predominantly reported in minced cartilage implantation

## Fibrin and thrombin as an autologous carrier matrix

Fibrin is a high-molecular-weight, non-water-soluble protein that is formed from fibrinogen (clotting factor I) during blood clotting by the enzymatic action of thrombin. Fibrinogen is a component of plasma. This can be easily produced, as described in the next section. There are medical devices into which PRP can be introduced to carry out the process of converting fibrinogen into fibrin. Thus, autologous fibrin glue can be produced in a short time. Allogeneic fibrin glue usually consists of human fibrinogen and other synthetic components which induce cross-linking of the fibrin.

In the arthroscopic minced cartilage technique, the chondrons are inserted into the defect and can be fixed with fibrin glue [61]. Fibrin sealants are commonly applied in medicine because of their complete biodegradable property. The mechanical strength of fibrin is low; therefore, it is often combined with scaffolds. The adhesive properties of fibrin are explored in example neurosurgery or visceral surgery for the repair of nerves or lesions of visceral organs such as the liver. In orthopedics, these products are used for the fixation of tissues such as tendons or even cartilage cells [68].

Fibrin sealants can be either allogenic or autologous. In allogenic fibrin sealants, human sealer protein and human thrombin combine into fibrin that adheres to human tissue. The fibrinogen concentration in these products is higher than the physiological levels, thus the resulting clot has a higher density than autologous products [27]. Autologous sealants can be prepared from whole blood or concentrated blood fractions such as platelet-rich (PRP) or platelet-poor plasma (PPP). In the coagulation cascade, fibrinogen (factor I) is combined with thrombin (IIa) to form fibrin (Ia). The fibrin can then form a cross-linked clot [65]. If the fibrin clot is prepared from autologous blood, a certain amount of growth factors is present, which is advantageous for the fixation of cartilage cells [17]. Unfortunately, however, the higher density of allogenic sealants also creates a more compact tissue which makes it more difficult for cells to migrate through the resulting tissue. This is disadvantageous, as the migration of growth factors or MSCs through the tissue is a requirement for the formation of a hyaline cartilage layer [33]. The autologous fibrin sealants have a significantly lower final density compared to allogeneic sealants. Interestingly, chondrocytes show a higher migration, proliferation, and matrix differentiation potential in autologous fibrin sealants [27]. Irwin et al. [39] were able to demonstrate the mechanical stability of an autologous fibrin clot. The mechanical properties of allogenic and all-autologous sealants were assessed. They compared the properties of sealants with PPP and PRP fibrinogen and allogenic sealants. The PPP and allogenic products were equally stable against axial and shear forces, the PRP product also showed stability, but this was less than in comparison to other components, caused by a faster clot formation. With PPP products the building of a clot takes longer so that the fluid that has not yet adhered can penetrate deeper into the tissue. Nevertheless, there is no current evidence that a deeper penetration leads to a higher regenerate quality. From the author’s experience with 250 patients, the stability of a PRP clot is sufficient since patients protect the joint from extraneous shear forces in the immediate post-operative period.

## Summary fibrin and thrombin as an autologous carrier matrix

- Both autologous and allogenic fibrin sealants are available
- Fibrin is completely biodegradable but has a low stability
- Autologous fibrin contains growth factors, which have the biologic potential to enhance chondrogenesis
- Cell migration is reported to be superior in autologous fibrin sealants compared to allogenic procedures
- PPP may penetrate deeper into the tissue compared to PRP

## PRP to augment cartilage repair

Cartilage metabolism requires a biological balance of anabolic and catabolic factors. It is known that PRP products can influence this tissue regulation [41]. PRP is produced by centrifugation of whole blood, which causes plasma to separate from the red blood cells. This process damages the cell membranes of red blood cells (RBC) and releases toxic hemoglobin, plasma-free hemoglobin, and iron. These products have a cytotoxic effect on tissues leading to oxidative stress, immunosuppression, and the activation of inflammatory pathways. Thus, the RBCs separated by centrifugation are discarded and only the plasma is used [60]. The plasma contains platelets which are an important element of biologic stimulation techniques. Centrifugation creates a platelet concentration that is above the whole blood baseline.

The effect of PRP on inflammation is important, as inflammatory processes can be accompanied by cartilage damage. PRP is thought to reduce inflammatory processes through several factors, including NF-κB, nitric oxide, and several chondrogenic growth factors [41]. Furthermore, PRP contains multiple growth factors that can stimulate the chondrogenic capacity of MSCs [30, 78]. Thus, multiple components of PRP can negatively influence the level of pro-inflammatory cytokines and matrix metalloproteinases (MMPs), in turn inhibiting cartilage degradation [21].

Another property of PRP is pain reduction. Serotonin (5-HT) is present on the platelets, which can interact with nociceptors and thus reduce pain. This 5-HT mechanism corresponds to the endogenous response to tissue injury or surgical trauma for the reduction of pain [64].

In minced cartilage technique, the harvested cartilage cells can be drizzled with PRP before implantation in the arthroscopic minced cartilage technique. PRP creates a fibrin gel including growth factors that can be released during degradation. This bio-scaffold created with the PRP enables minced cartilage fragment integration [75]. The effects of PRP remain controversial, however. Olesen et al. [55] performed an in vivo study in which he performed MCI for chondral defects in 6 Göttingen- mini-pigs. These authors found that repeated local injection of PRP did not provide beneficial effects in the macroscopic and histologic evaluation of the resulting repair tissue. Alternatively, Cugat et al. [23] reported technical outcomes of minced cartilage with a bio-matrix composed of PRP and plasma rich growth factor (PRGF). Preliminary results of 15 patients demonstrated excellent clinical, functional and radiographic outcomes in 16 months of follow-up.

Though a commonly known technique, a definitive standardized protocol for PRP formulation does not exist. Protocols of commercial PRP preparation systems vary in the dosage, choice of anticoagulants to prevent clotting, spin rate, and supplementation of other factors [21]. These variables may greatly influence the outcomes of PRP and the ability to study its use. For example, Sabarish et al. [58] demonstrated that different spin rates had a significant impact on platelet yields.

The biologic potential of PRP specifically in MCI is not extensively investigated, thus, while preliminary data supports that the application of a full autologous approach using PRP as bio-matrix is safe and may provide a positive environment for chondrogenic proliferation and differentiation of activated chondrocytes, more research is needed in this area.

## Summary PRP to augment cartilage repair

- PRP has a high regenerative ability with the potential to reduce inflammation, induce angiogenesis, proliferation, and stem cell migration
- PRP provides a high number of growth factors and potential for inhibition of catabolic processes (e.g., TNFα, IL-1β)
- PRP clot enables tissue integration as bio-scaffold
- Preliminary data in MCI demonstrate PRP is safe and has beneficial potential for cartilage regeneration

## Joint homeostasis before and after cartilage repair

Injured cartilage is characterized by disturbed joint homeostasis with inflammation and reduced expression of extracellular matrix components [35]. This disturbed joint homeostasis can result in the progression of osteoarthritis, affecting the whole joint. The release of inflammatory cytokines such as tumor necrosis factor α (TNFα) is an important trigger of early osteoarthritis and is a proposed biomarker candidate to monitor osteoarthritic progression [15]. It has been shown previously that antagonization of inflammatory cytokines and mechanical stimulation of degenerative cartilage reduces osteoarthritic processes and stimulates chondrogenesis [69]. However, *restitutio ad integru*m—restoration to original condition—was not reached in these studies, making cartilage replacement therapies essential for the treatment of focal, degenerative, and severely lesioned and eroded hyaline cartilage to prevent further osteoarthritic progression in joints. As such, it has been shown by data from a German cartilage registry that a majority (about 60%) of autologous cartilage implantations were performed in patients with degenerative, non-traumatic cartilage lesions [54]. A significant percentage of ACI graft failures occur in degenerative joints and ongoing inflammatory processes are thought to contribute to graft failure [5].

It remains to be investigated whether immunomodulatory therapy after cartilage transplantation/implantation would result in fewer failures [56]. Condello et al. reported a therapy with biomimetic scaffolds in 26 patients with focal early osteoarthritis with type I equine collagen and magnesium-enriched hydroxyapatite [22]. Clinical outcomes improved in 69% of patients without disease-modifying effects. Sessa et al. demonstrated a 3-years follow-up of 22 patients with focal OA treated with tri-layered nanostructured biomimetic osteochondral scaffolds with a promising clinical outcome. On top, a biological and biomechanical approach including cell-free scaffold constructs, osteotomy, and meniscal implants showed good clinical results in patients affected by symptomatic unicompartmental OA [48].

Minced cartilage implantation seems to positively stimulate the local “cytokinetic climate” as implanted cartilage is well integrated into the surrounding tissue by outgrowing and matrix-depositing chondrocytes reported in an in-vivo goat model [50]. In addition to an optimal chemical environment, mechanical stimulation is an important factor for cartilage regeneration after surgery. Wang et al. [72] investigated the regenerative capacity of cartilage fragments and passaged chondrocytes in a knee joint-specific bioreactor. Compression and shear led to an increased matrix production and a chondrogenic gene expression pattern. Mechanical processing of implanted cartilage (including mincing of cartilage before re-implantation) has been shown to promote the production of functional ECM. The rehabilitation after cartilage repair is important for the regeneration of the repair tissue. MCI rehabilitation protocol is following the principles after ACI [61]. Further research is necessary to focus on the regenerative effect of MCI in osteoarthritic surroundings.

## Summary joint homeostasis before and after cartilage repair

- Cartilage repair is often performed in degenerative surroundings
- The reduction of inflammatory processes may support cartilage regeneration
- Physiologic mechanical stimulation improves cartilage homeostasis
- Minced cartilage tissue integration may support regenerative potential in early OA

## Conclusion

Particulated autologous cartilage has a strong biologic potential and holds good promise to repair such lesions effectively and durably. It is an autologous procedure and can be placed in a one-step surgery using arthroscopic techniques. Chondrocytes are mechanically activated via mincing, transplanted chondrocytes with surrounding ECM and PCM (chondron) can proliferate/differentiate in situ under physiobiomechanical chondrogenic surroundings within the joint. While there is strong in vitro and preclinical evidence for minced cartilage implantation, the translation into clinics remains limited. Further standardization of the procedure and clinical studies are required to fully develop and understand the biology and implantation techniques using minced cartilage implantation to maximize clinical outcomes.
